# LDHB Overexpression Can Partially Overcome T Cell Inhibition by Lactic Acid

**DOI:** 10.3390/ijms23115970

**Published:** 2022-05-26

**Authors:** Sonja-Maria Decking, Christina Bruss, Nathalie Babl, Sebastian Bittner, Sebastian Klobuch, Simone Thomas, Markus Feuerer, Petra Hoffmann, Katja Dettmer, Peter J. Oefner, Kathrin Renner, Marina Kreutz

**Affiliations:** 1 LIT—Leibniz Institute for Immunotherapy, 93053 Regensburg, Germany; sonja.decking@gmail.com (S.-M.D.); sebastian.bittner@ukr.de (S.B.); simone.thomas@ukr.de (S.T.); markus.feuerer@ukr.de (M.F.); petra.hoffmann@ukr.de (P.H.); kathrin.renner-sattler@ukr.de (K.R.); 2 Department of Internal Medicine III, University Hospital Regensburg, 93053 Regensburg, Germany; christina.bruss@ukr.de (C.B.); nathalie.babl@ukr.de (N.B.); 3 Department of Gynecology and Obstetrics, University Hospital Regensburg, 93053 Regensburg, Germany; 4 Department of Medical Oncology, The Netherlands Cancer Institute, Plesmanlaan 121, 1066 CX Amsterdam, The Netherlands; s.klobuch@nki.nl; 5 Institute of Functional Genomics, University of Regensburg, 93053 Regensburg, Germany; katja.dettmer@ukr.de (K.D.); peter.oefner@ukr.de (P.J.O.)

**Keywords:** T cells, lactic acid, glycolysis, LDH, interferon gamma, adoptive cell transfer, lactate, T cell metabolism

## Abstract

Accelerated glycolysis leads to secretion and accumulation of lactate and protons in the tumor environment and determines the efficacy of adoptive T cell and checkpoint inhibition therapy. Here, we analyzed effects of lactic acid on different human CD4 T cell subsets and aimed to increase CD4 T cell resistance towards lactic acid. In all CD4 T cell subsets analyzed, lactic acid inhibited metabolic activity (glycolysis and respiration), cytokine secretion, and cell proliferation. Overexpression of the lactate-metabolizing isoenzyme LDHB increased cell respiration and mitigated lactic acid effects on intracellular cytokine production. Strikingly, LDHB-overexpressing cells preferentially migrated into HCT116 tumor spheroids and displayed higher expression of cytotoxic effector molecules. We conclude, that LDHB overexpression might be a promising strategy to increase the efficacy of adoptive T cell transfer therapy.

## 1. Introduction

Adoptive cell transfer therapy (ACT) using either patient-derived T cell clones or T cells equipped with chimeric antigen receptors (CARs) has shown great promise in the treatment of hematological malignancies, but thus far limited efficacy against solid tumors [1]. Potential obstacles include immunosuppressive factors that render T cells dysfunctional and low T cell infiltration and/or apoptosis in the tumor microenvironment [2]. Cascone and colleagues have demonstrated that the response of melanoma patients to ACT is negatively correlated to the expression of glycolytic genes in the tumor tissue [3]. Therefore, tumor glycolysis-related immune escape mechanisms seem to limit the efficacy of ACT.

Tumor glycolysis, the so-called “Warburg effect”, represents a well-known metabolic feature in the tumor environment [4,5]. Here, enhanced glucose metabolism of tumor cells results in secretion of lactate and protons via monocarboxylate transporters (MCTs) leading to acidification of the tumor microenvironment. In metastasized melanoma, lactate levels range between 5 mM and 40 mM [6]. Lactate accumulation correlates with both, a poor overall survival or tumor recurrence e.g., in cervix and head-and-neck cancer (HNSCC) [7,8], and the incidence of metastasis in cancer of the rectum, cervix and HNSCC [7,9,10,11]. One possible explanation for the linkage between the Warburg effect and worse prognosis is the suppression of an effective anti-tumor immune response by lactic acid [12].

Several studies have investigated the effects of lactic acid on the functional activity of different types of immune cells. Lactic acid treatment inhibits cytokine production and differentiation of human monocytes [13] and limits antigen presentation and migration of dendritic cells [14,15]. Moreover, lactic acid decreased cytokine production by NK and T cells and induced cell death at higher concentrations [6,16,17,18]. In line, disrupting the capability of tumor cells to produce and secrete lactate by genetic or pharmacological intervention increases the immunological anti-tumor response [6,19,20,21] and enhances the efficacy of checkpoint blockade therapy [22,23,24], supporting a direct link between tumor glycolysis, lactic acid accumulation in the tumor environment and immune evasion. 

Immunosuppression by lactic acid is related to the metabolic similarities and interplay between tumor and immune cells. Upon activation, T cells upregulate glycolysis and oxidative phosphorylation (OXPHOS), which is essential for proliferation and cytokine production [25]. The export of lactate by MCTs depends on a gradient from cytoplasmic to extracellular lactate concentrations. However, high extracellular concentrations of lactic acid in the tumor lead to lactate and proton uptake by T cells and, subsequently, lower their intracellular pH [6,26], which in turn slows down metabolic activity and activation. In line with these findings, tumor-infiltrating lymphocytes are often characterized by mitochondrial and glycolytic dysregulation in murine and human tumors [27,28,29]. In addition, the decrease in response of chronic lymphatic leukemia (CLL) patients to CAR T cell therapy is related to mitochondrial impairment of the infused CAR T cells [30]. Therefore, enhancing T cell metabolic fitness seems a promising strategy to support ACT.

Here, we analyzed the effects of lactic acid on human CD4 T cells and tried to increase their lactic acid resistance by manipulating the lactate dehydrogenase (LDH) isoenzyme balance. LDH catalyzes the interconversion of lactate and pyruvate. The enzyme is a tetramer composed by two subunits, LDHA and LDHB, resulting in five different LDH isoenzymes (LDH-1 to LDH-5). It has been described that LDHA has a higher affinity towards pyruvate as a substrate whilst LDHB has a higher affinity for lactate [31]. We hypothesized that overexpression of LDHB could counteract the adverse effects of lactate and proton influx in T cells via metabolic conversion of lactate to pyruvate. LDHB overexpression indeed enhanced both, T cell respiration and cytokine production in the presence of up to 15 mM lactic acid. 

## 2. Results

### 2.1. Subtype Independent Inhibition of CD4 T Cell Effector Function and Metabolism by Lactic Acid 

CD4 T cells are important for the development and sustainment of a T cell mediated anti-tumor response. As previously shown for murine and human CD8 T cells [6,18], treatment with 20 mM lactic acid also significantly decreased cell viability of bulk human CD4 T cells (Figure S1A). Lower concentrations of lactic acid diminished cell proliferation and effector cytokine secretion (Figure S1B,C) as well as oxygen and glucose consumption (Figure S1D,E). Moreover, treatment with 15 mM lactic acid prevented upregulation of LDHA upon activation (Figure S1G), indicating a disturbed glucose metabolism. 

**Figure 1:**
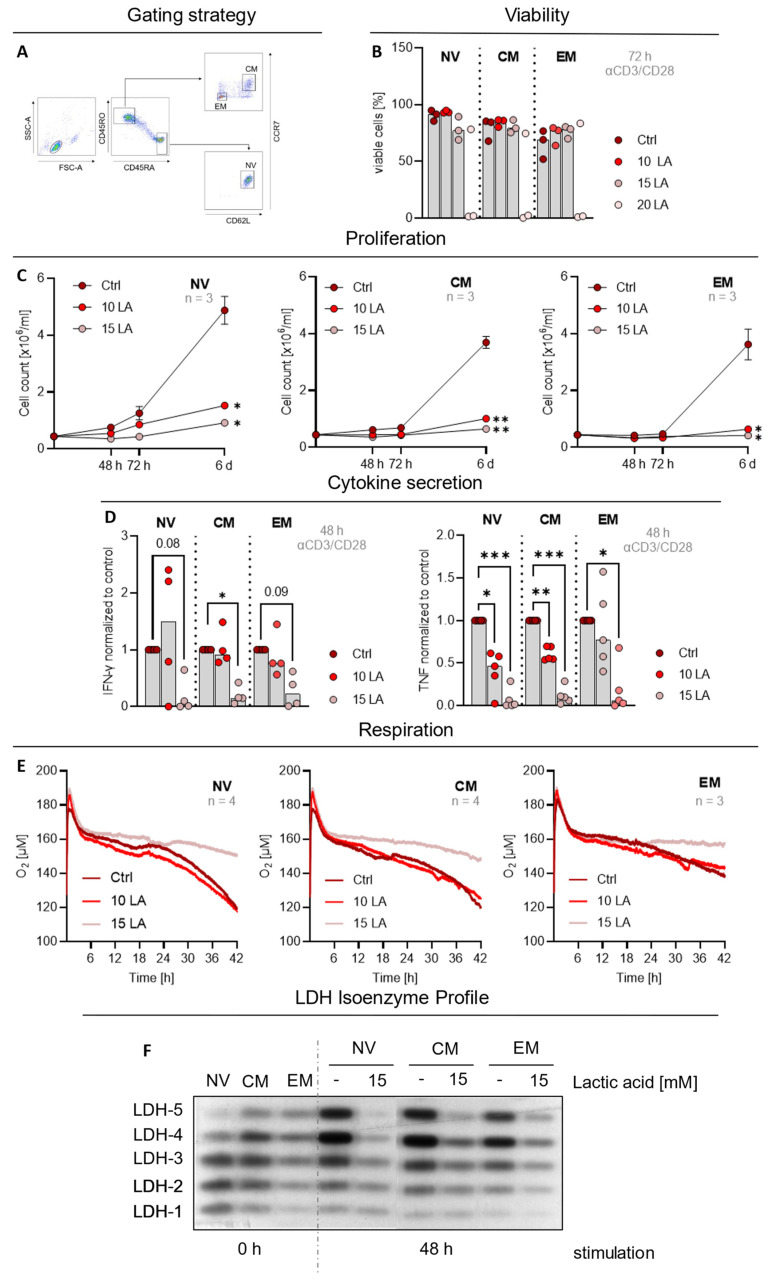
CD4 T cell subsets do not show inherent resistance towards lactic acid. (**A**) Representative gating strategy for cell sorting of naïve (NV), central memory (CM) and effector memory (EM) cells by flow cytometry. (**B**–**F**) Naïve (NV), central memory (CM), and effector memory (EM) CD4 T cells were sorted by flow cytometry. Subsets were stimulated with anti-CD3/CD28 coated beads (bead-to-cell ratio 1:1, except for (**F**) and treated with the indicated concentrations of lactic acid (LA). (**B**) Viability was determined by flow cytometry analysis of the FSC/SSC pattern after 72 h. Shown are median values and single data points. Statistical significance was calculated using one-way ANOVA and Bonferroni multiple comparison test (no significance detected). (**C**) Cells were counted at indicated time points using the CASY Cell Counter (mean ± SEM, *n* = 4). Statistical significance was calculated with two-way ANOVA and Dunnett’s multiple comparison test (* *p* < 0.05; ** *p* < 0.01). (**D**) Cytokine concentrations in supernatants were determined by ELISA after 48 h and normalized to the respective controls. Shown are median values and single data points. Statistical significance was calculated using one-way ANOVA and Bonferroni multiple comparison test (* *p* < 0.05, ** *p* < 0.01, *** *p* < 0.001; digits give exact *p* values). (**E**) Oxygen consumption of the cells was measured using the PreSens technology (mean values, *n* = 3–4). (**F**) LDH isoenzyme distribution of unstimulated and cells stimulated for 48 h in the presence or absence of lactic acid was assessed using LDH zymography. Depicted is one representative example (unstimulated cells: *n* = 4, stimulated controls: *n* = 3, lactic acid treated cells: *n* = 2).

Different studies have demonstrated that a successful response to ACT strongly depends on the memory state of the transferred CD8 T cells [32,33,34]. We hypothesized that this might be due to higher lactic acid-resistance of memory T cells. Therefore, we used flow cytometry to sort naïve (NV), central memory (CM) and effector memory (EM) CD4 T cells (Figure 1A) and analyzed the effects of lactic acid. As fluorochrome-labelled sorting antibodies remain bound to the cell surface, viability analysis by Annexin V/7-AAD was not possible in sorted cells. Instead, viability was determined by analysis of the forward- and side scatter characteristics. Comparable to bulk CD4 T cells, 20 mM lactic acid treatment resulted in cell death induction in two of three analyzed donors after 72 h (Figure 1B) and cell proliferation was significantly inhibited after treatment with 10 mM and 15 mM lactic acid, respectively, regardless of the analyzed subpopulation (Figure 1C). Furthermore, 15 mM lactic acid decreased IFN-γ secretion only in CM significantly but not in stimulated naïve and EM T cells (Figure 1D), whereas TNF secretion (Figure 1D) and respiratory activity (Figure 1E) were lowered in all CD4 T cell subsets. However, in EM cells two out of four donors were not influenced by lactic acid. Lastly, we analyzed the influence of lactic acid treatment on LDH isoenzyme after activation by performing LDH zymography. This technique allows the separation and visualization of the different LDH isoenzymes. The basal LDH isoenzyme pattern was strikingly different between the CD4 subsets. Naïve T cells showed the lowest and EM T cells the highest levels of LDH-5, the homotetramer of LDHA (Figure 1F), possibly reflecting differences in glycolytic activity and respiration. However, upon activation, all subsets upregulated LDHA, inducing a stronger shift towards LDH-5, which was blocked by lactic acid treatment (Figure 1F). Overall, lactic acid treated T cells exhibited less LDH protein as the zymography band intensity was reduced despite equal protein loading. In summary, we were unable to identify a CD4 memory subset with complete lactic acid resistance.

Metabolic inhibition, especially of T cell glycolysis, accounts for major parts of the lactic acid effects [18,26]. Transient glucose restriction in murine CD8 T cells prior to ACT has been shown to enhance the in vivo anti-tumor response and tumor control [35]. Therefore, we hypothesized, that glucose restriction would probably limit MCT upregulation upon T cell activation [23], thereby circumventing lactic acid influx and glycolytic inhibition leading to higher lactic acid resistance. We cultivated CD4 T cells with different glucose concentrations while treating them with 15 mM lactic acid. Glucose restriction did not increase oxygen consumption of T cells (Figure S2A) or alter the LDH isoenzyme profile (Figure S2B). Regardless of the glucose concentration, treatment with 15 mM lactic acid blocked cellular respiration and the shift in the LDH isoenzyme pattern. As described before, glucose restriction decreased the proliferative activity of T cells (Figure S2C) and the remaining capacity for T cell proliferation was blocked by lactic acid treatment. In addition, 15 mM lactic acid inhibited secretion of IFN-γ and TNF (Figure S2D,E). Overall, these results demonstrate that glucose restriction does not increase lactic acid resistance of human CD4 T cells.

### 2.2. LDHB Overexpression in T Cells Increases Basal Respiration but Does Not Influence Cytokine Secretion and Polarization 

**Figure 2:**
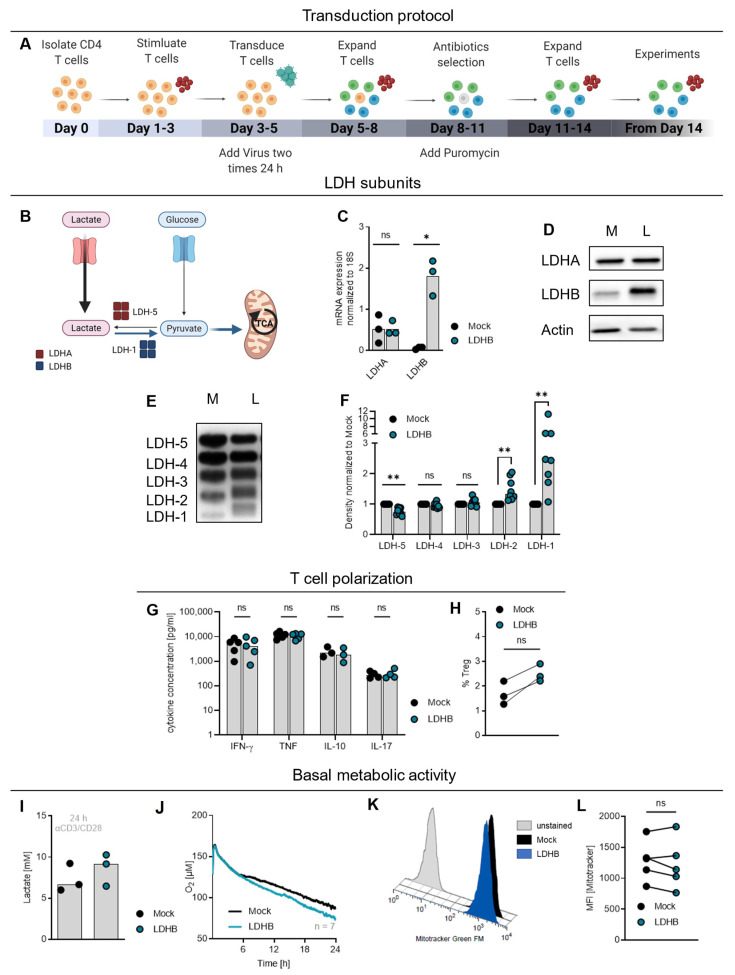
LDHB overexpression in human CD4 T cells does not alter cytokine profile or lactate secretion, but slightly increases cellular respiration. (**A**) Schematic overview over the retroviral transduction process to overexpress LDHB. Red dots represent Dynabeads. Graphic created with BioRender.com. (**B**) Schematic depiction of the impact of LDHB overexpression in lactic acid treated T cells. Graphic created with BioRender.com. (**C**–**K**) LDHB was overexpressed in human CD4 T cells. Empty vector transduced T cells (Mock) served as control. (**C**) Expression of LDHA and LDHB mRNA was determined by quantitative RT-PCR and normalized to 18S rRNA. Depicted are median levels and single data points. Statistical significance was calculated with Wilcoxon’s matched pair signed rank test (ns not significant, * *p* < 0.05). (**D**) Western blot analysis of LDHA and LDHB in Mock (M) and LDHB (L) transduced cells. Actin served as loading control. Shown is one representative example (*n* = 5). (**E**) LDH isoenzyme distribution in Mock (M) and LDHB (L) transduced cells. Given is one representative example (*n* = 8). (**F**) Band intensities of the LDH isoenzyme analysis given in (**E**) were quantified using ImageJ and normalized to the respective Mock value. Depicted are median levels and single data points. Statistical significance was calculated with Wilcoxon’s matched pair signed rank test (ns not significant, ** *p* < 0.01). (**G**) Cells were stimulated with PMA/Ionomycin for 3 h. Cytokine levels in supernatants were determined by ELISA. Depicted are median levels and single data points. Statistical significance was calculated with Wilcoxon’s matched pair signed rank test (ns not significant). (**H**) Portion of regulatory T cells (Treg; FOXP3^high^ CD25^high^) within Mock and LDHB transduced cells was determined by flow cytometry. Statistical significance was calculated with Wilcoxon’s matched pair signed rank test (ns not significant). (**I**,**J**) Cells were stimulated with anti-CD3/CD28 coated beads (beads-to-cell ratio 1:1). (**I**) Lactate concentration in the supernatants was measured enzymatically after 24 h. (**J**) Oxygen consumption of the cells was assessed using the PreSens technology. (**K**,**L**) Mitochondrial content was determined by staining with the fluorescent dye Mitotracker Green FM and subsequent flow cytometry analysis. Depicted are (**K**) one representative example and (**L**) median fluorescence intensity of single data points. Lines connect paired values. Statistical significance was calculated with Wilcoxon’s matched pair signed rank test (ns not significant).

LDH inhibition was shown to increase T cell respiratory activity and enhance their anti-tumor effects in vivo [36]. Therefore, we hypothesized that direct manipulation of the LDH isoenzyme balance could have a similar effect on cellular metabolism and increase T cell lactic acid resistance. By shifting the LDH isoenzyme profile towards LDHB and enhancing the proportion of the LDHB homotetramer LDH-1 by retroviral transduction (Figure 2A), we aimed to endow the T cells with the capacity to metabolize lactate, thereby enhancing cellular respiration and mitigating lactic acid effects (Figure 2B).

In a first approach, we downregulated LDHA in CD4 T cells by siRNA electroporation and analyzed how this manipulation would influence the T cell LDH isoenzyme pattern. In unstimulated T cells, the LDH isoenzyme profile T cells was not altered by siLDHA treatment (Figure S3A) but a clear shift towards LDHB containing isoenzymes was detected after 48 h activation. After 6 days, the effect of siLDHA electroporation on LDH isoenzyme profile was no longer detectable. Surprisingly, neither oxygen consumption nor lactate production were altered in siLDHA-treated cells (Figure S3B,C). Moreover, LDHA downregulation did not increase T cell lactic acid resistance in terms of cytokine production (Figure S3D,E). As the electroporation itself seemed to cause a strong decline in respiratory activity and cytokine production (data not shown), we concluded that this method is not suitable for further studies.

Alternatively, we decided to shift the LDH isoenzyme profile by overexpression of LDHB and retrovirally transduced CD4 T cells with a HIS-tagged LDHB. Empty-vector transduced T cells (Mock) served as control. After retroviral transduction of T cells, a selection with puromycin was performed to ensure population purity (schematic depiction of the protocol in Figure 2A). LDHB was strongly overexpressed on mRNA and protein level, whereas expression of LDHA was not altered (Figure 2C,D). This was reflected in the LDH zymography analysis (Figure 2E,F), which revealed an increased portion of the LDH-containing isoform LDH-1 and LDH-2, but a decreased amount of LDH-5 in LDHB T cells. This indicates, that LDHA is redistributed to pair with overexpressed LDHB subunits leading to a LDH isoenzyme shift.

A relationship between T cell differentiation and cellular metabolism has been demonstrated in murine T cells [37,38,39,40]. Therefore, we analyzed whether LDHB overexpression would alter CD4 T cell differentiation. CD4 T cells may differentiate into distinct T helper (Th) cell populations or induced regulatory T cell (Treg) characterized by different cytokine profiles. However, secretion of IFN-γ and TNF, both signature cytokines of Th1 cells, were not altered by LDHB overexpression. Similar results were found for IL-10, a signature cytokine of Th2 and Treg, and IL-17, a signature cytokine of Th17 cells (Figure 2G). The percentage of FOXP3^high^ CD25^high^cells, considered as induced Treg, was slightly increased among the LDHB T cells but the overall percentage of Treg in the culture remained low (Figure 2H). Overall LDHB overexpression did not alter T cell differentiation.

Finally, we investigated how T cell metabolism was influenced by LDHB overexpression. Lactate secretion within 24 h after activation was not altered in LDHB T cells (Figure 2I). Cellular oxygen consumption was slightly increased in LDHB overexpressing T cells (Figure 2J) compared to Mock cells starting 6 h after activation at equal mitochondrial content (Figure 2K,L), indicating an increased mitochondrial activity of LDHB T cells. 

### 2.3. Effect of LDHB Overexpression on Cytokine Production and Cell Metabolism upon Lactic Acid Treatment

**Figure 3:**
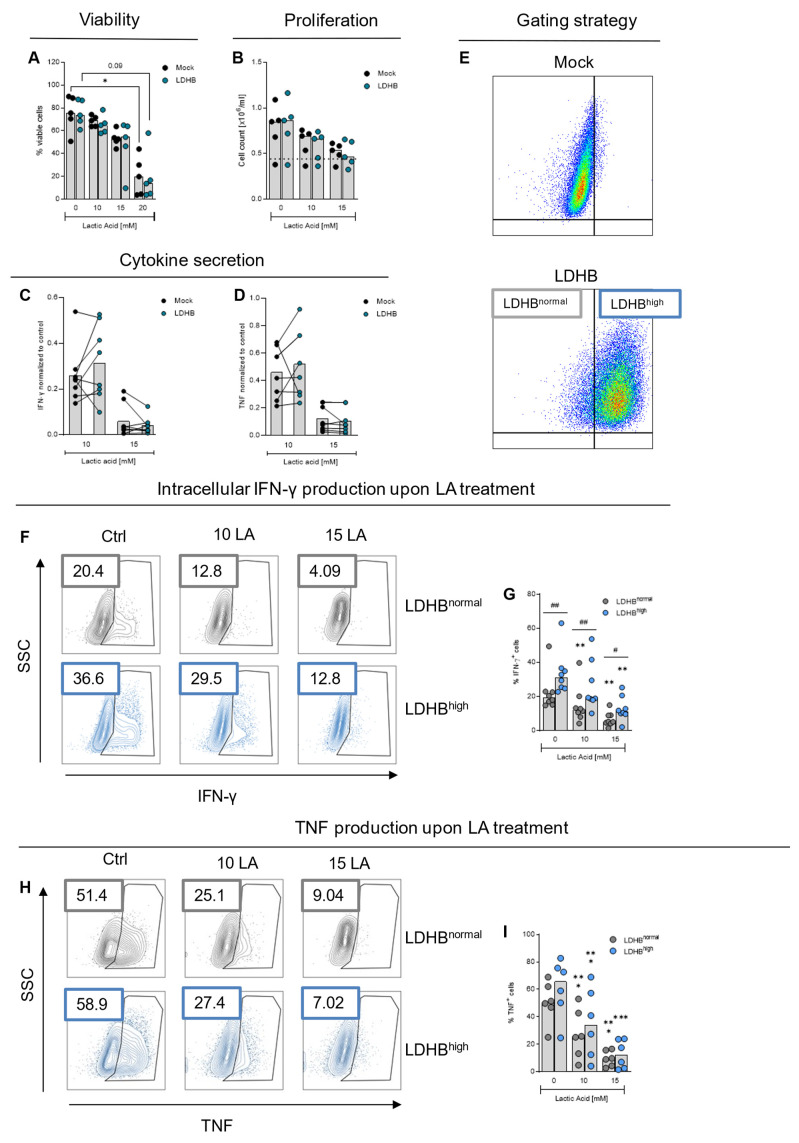
LDHB overexpression in human CD4 T cells mitigates lactic acid effect on cytokine production. (**A**,**B**) Cells were stimulated with α-CD3/CD28 coated beads in the presence of the indicated concentrations lactic acid. (**A**) Cell viability was assessed by Annexin V/7-AAD staining after 48 h stimulation. (**B**) Cells were counted after 24 h stimulation. Dotted lines represents seeded cell number (0.44 × 10^6^/mL). (**C**–**I**) Cells were pre-incubated with the respective treatments overnight and then stimulated with PMA/Ionomycin for three hours. (**C**,**D**) Concentrations of (**C**) IFN-γ and (**D**) TNF in culture supernatants were determined by ELISA and normalized to the respective control. Lines connect linked data points. (**E**–**I**) For intracellular cytokine staining, cells were treated with monensin. (**E**) Representative FACS-plots depicting the anti-HIS staining and gating for panel (**D**–**G**). X-Axis: HIS; Y-Axis: SSC. (**F**–**I**). Cells were stained for (**F**,**G**) IFN-γ and (**H**,**I**) TNF and separated according to HIS expression. Given is (**F**,**H**) one representative analysis and (**G**,**I**) median levels and single data points. Statistical significance was calculated with one-way ANOVA and Bonferroni multiple comparison test. Asterisks give significance compared to respective control, hashes within one lactic acid concentration between Mock and LDHB (* *p* < 0.05, ** *p* < 0.01, *** *p* < 0.001; same for #).

Next, we investigated whether LDHB overexpression would increase lactic acid resistance of CD4 T cells. We treated Mock and LDHB overexpressing T cells with up to 20 mM lactic acid and assessed influence on viability (Figure 3A) and cell proliferation (Figure 3B). As observed in primary stimulated T cells, lactic acid treatment induced cell death in higher concentrations (Figure 3A) and reduced cell proliferation (Figure 3B). Both effects were not influenced by LDHB overexpression in our analysis.

Although not increasing the resistance of T cells towards fatal concentrations of lactic acid, increasing the lactic acid resistance in terms of cytokine secretion could lead to better T cell activity in highly glycolytic environments. Therefore, we measured the concentrations of IFN-γ and TNF in the supernatants of Mock and LDHB overexpressing cells after stimulation with PMA/Ionomycin and treatment with different concentrations of lactic acid (Figure 3C,D). Diminished inhibition of cytokine secretion was observed in four of eight analyzed donors upon treatment with 10 mM lactic acid. Upon treatment with 15 mM lactic acid, LDHB overexpression had no beneficial effect on cytokine secretion, indicating that 15 mM lactic acid confers an effect too strong to be rescued by LDHB overexpression. 

Despite being HIS positive to 95–98% after selection, some cells lose expression of the HIS-tagged LDHB after selection (LDHB^normal^). We wondered whether this phenomenon would explain the heterogeneous effect on cytokine secretion. Therefore, we used the HIS-Tag to distinguish between LDHB overexpressing (HIS positive, LDHB^high^) cells and cells with normal LDHB levels (HIS negative, LDHB^normal^) within the LDHB transduced cells (gating strategy in Figure 3E) and analyzed intracellular cytokine production (Figure 3F–I). Among HIS positive cells we found a significant increase in IFN-γ producing cells, which was also detected upon lactic acid treatment, indicating a beneficial effect of LDHB on cytokine production (Figure 3F,G). This effect was less pronounced and donor-dependent regarding TNF expression (Figure 3H,I). 

Next, we were interested in why LDHB overexpression supports cytokine production in CD4 T cells. Upon activation, T cells upregulate several metabolic pathways to fuel cell proliferation and cytokine production and several studies have suggested a direct link between effector functions and T cell glucose metabolism. Therefore, we analyzed how the cellular metabolism of T cells was influenced by LDHB overexpression upon lactic acid treatment. 

**Figure 4:**
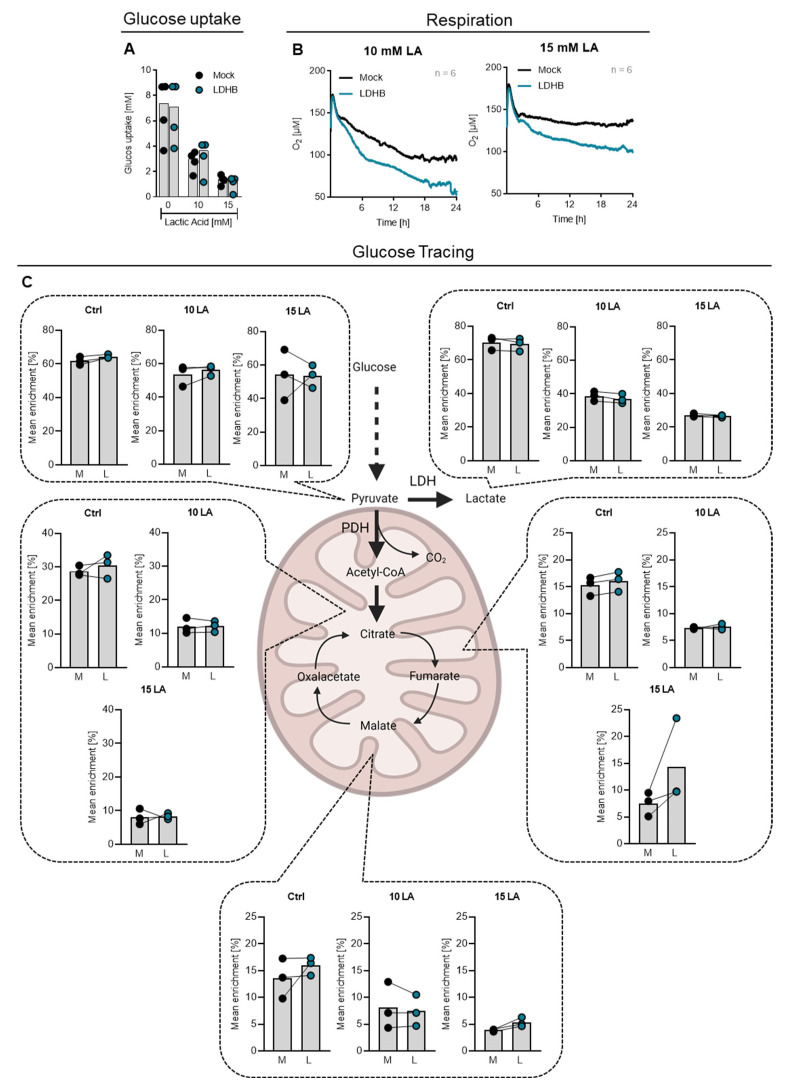
LDHB overexpression in human CD4 T cells increases cellular respiration upon lactic acid treatment but does not alter glucose metabolism. (**A**) Cells were stimulated with anti-CD3/CD28 coated beads (bead-to-cell ratio 1:1) and treated with the indicated concentrations of lactic acid for 24 h. The remaining glucose in the supernatant was measured photometrically and subtracted from the medium glucose concentration to calculate glucose uptake. Shown are median levels and single data points. Significance was calculated using one-way ANOVA and Bonferroni multiple comparison test (no significance detected). (**B**) Cells were stimulated with anti-CD3/CD28 coated beads (bead-to-cell ratio 1:1), treated with lactic acid as indicated and oxygen consumption was monitored using the PreSens technology (mean values, *n* = 6). (**C**) Cells were stimulated with anti-CD3/CD28 coated beads (bead-to-cell ratio 1:5), incubated with 10 mM U13C-Glucose and treated with lactic acid as indicated. After 6 h, cells were lysed and distribution of the 13C label was measured by mass spectrometry. Depicted are median levels and single data points. Lines connect paired samples. Significance was calculated using Wilcoxon’s matched-pair signed rank test (no significance differences between Mock and LDHB overexpressing cells). Schematic depiction of glucose metabolism and citrate cycle created with BioRender.com.

As observed in primary stimulated T cells, lactic acid treatment distinctly lowered glucose consumption of T cells independent of LDHB overexpression (Figure 4A). However, analyzing cellular respiration, we found that LDHB overexpressing cells showed an increased oxygen consumption upon lactic acid treatment, revealing an increased mitochondrial activity (Figure 4B).

The tricarboxylic acid cycle (TCA) in mitochondria, which is responsible for the generation of respiratory substrates, is fueled by different pathways. First, glucose can be metabolized to pyruvate, which is further metabolized to acetyl-CoA by pyruvate dehydrogenase (PDH) and introduced into TCA. In addition, pyruvate can be generated from lactate which is converted to pyruvate by LDH activity.

Since we manipulated LDH, we hypothesized that LDHB might directly influence the T cells’ glucose usage. In order to trace cellular glucose metabolism, we incubated T cells with uniformly 13C-labeled glucose. After 6 h, the enrichment of the 13C label in key metabolites was measured (Figure 4C). 

Isotope enrichment analysis revealed no decreased enrichment of the 13C label in pyruvate upon lactic acid treatment, indicating a preserved glucose flux up to this point. In contrast, 13C enrichment in lactate and TCA metabolites citrate, fumarate and malate were distinctly lowered in lactic acid treated cells. This could indicate that the metabolism of pyruvate by both LDH and PDH is blocked by lactic acid treatment irrespectively of LDB overexpression. 

However, an unchanged isotope enrichment does not exclude an overall elevated lactate metabolism in LDHB overexpressing cells, and the decreased 13C enrichment in lactate could be a consequence of cellular lactate uptake. Additionally, changes in glutamine or fatty acid metabolism may be responsible for higher respiratory activity in LDHB overexpressing cells which should be elucidated in further studies. 

### 2.4. LDHB Overexpressing T Cells Show Enhanced Infiltration and Cytotoxicity in HCT116 Spheroids

To the end, we investigated the anti-tumor activity of LDHB overexpressing T cells in an in vivo syngeneic tumor model. For this purpose, we overexpressed Ldhb in T cells isolated from C57BL/6 mice and tested their resistance towards lactic acid. 

Retroviral transduction led to a highly efficient and strong overexpression of Ldhb in CD4 T cells, as shown in Western blot analyses (Figure S4A). Analyzing the Ldh isoenzyme profile of the cells, we found major differences compared to human T cells: while human T cells upregulate LDHA upon stimulation, but still express all other isoenzymes, murine T cells express predominantly Ldh-5, the homotetramer of Ldha, and only a little amount of Ldhb containing isoforms (Figure S4B). A clear shift of the pattern was observed in Ldhb transduced cells, though the Ldha overload persisted. In line, murine T cells displayed only weak respiratory activity (Figure S4C) that could not be improved by Ldhb overexpression. Finally, no increase in cytokine expression or secretion was observed (Figure S4D).

As a consequence, we decided to use a human spheroid co-culture model to estimate the anti-tumor capacities of LDHB overexpressing cells. Spheroids are three-dimensional growing tumors resembling avascular small metastases. Similar to solid tumors, they display inwardly decreasing gradients of nutrients and oxygen and increasing concentrations of lactate and protons, making them a good approximation to a real tumor situation. 

**Figure 5:**
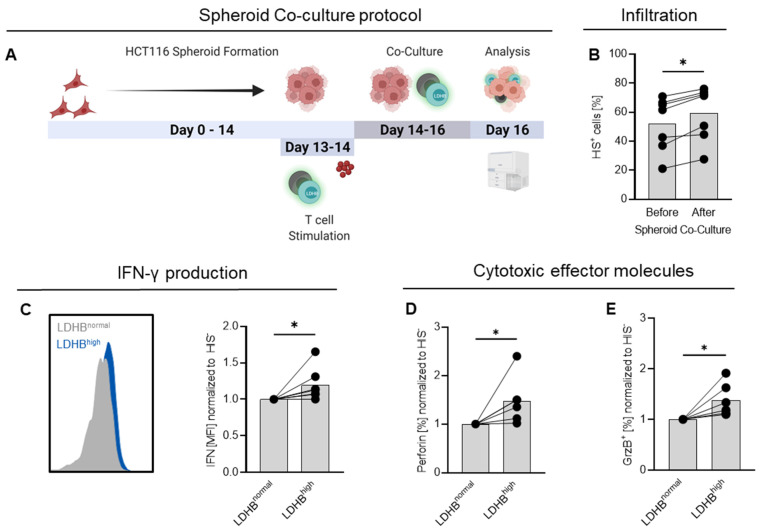
LDHB-overexpressing CD4 T cells show improved infiltration and higher expression of cytotoxic effector molecules in HCT116 spheroid co-cultures. (**A**) Schematic depiction of the co-culture protocol. Graphic created with BioRender.com. (**B**) Percentage of LDHB^high^ cells was assessed by flow cytometry before addition to spheroids and after co-culture among infiltrated T cells. (**C**–**E**) After 48 h co-culture, monensin was added to spheroid co-cultures. After 3 h, single cell suspensions were generated from co-cultures and T cells analyzed for their expression of (**C**) IFN, (**D**) Perforin and (**E**) Granzyme B (GrzB). The HIS-Tag was used for identification of LDHB overexpressing cells. For analysis, (**C**) median fluorescence intensity (MFI) and (**D**,**E**) percentage of positive cells measured among LDHB^high^ cells were normalized on LDHB^normal^ cells. (**B**–**E**) Shown are median levels and single data points. Lines connect paired data. Statistical significance was calculated using Wilcoxon’s matched pair signed rank test (* *p* < 0.05).

In this study, HCT116 spheroids were grown for 10–14 days before adding the genetically manipulated T cells. We used bulk transduced T cells not selected for LDHB expression, resembling a heterogeneous population of T cells with and without LDHB overexpression. This approach enabled us to study T cells with higher and lower LDHB levels in the same tumor co-culture in parallel. After 48 h of co-culture, non-infiltrated immune cells were washed away, spheroids were lysed, and the infiltrating immune cells were analyzed (schematic depiction of the protocol in Figure 5A). 

We assessed the percentage of HIS-expressing T cells before and after 48 h co-culture. In all analyzed donors, the portion of HIS-positive cells was higher among the infiltrating immune cells (Figure 5B), indicating the LDHB-overexpressing T cells preferentially infiltrated into the spheroid environment compared to non-transduced cells. Using the HIS-Tag to distinguish between LDHB-overexpressing and normal T cells, we also detected higher levels of IFN-γ and higher portions of granzyme B and perforin-expressing T cells among the HIS-positive cells, indicating that LDHB-overexpressing T cells may support cytotoxic activity within the HCT116 spheroids. Together, these data indicate that LDHB overexpression can improve T cell infiltration and function in a suppressive tumor environment.

## 3. Discussion

In addition to an abnormal and accelerated cell division and the ability to form metastases, a dysregulated cellular metabolism is one of the most important functional characteristics of cancer cells. Among different known metabolic alterations in cancer cells, the Warburg effect, representing the shift from an oxidative to a glycolytic metabolism, is found in the majority of human tumors. It goes along with enhanced glucose consumption and increased secretion of lactate and protons. Several studies have shown a link between enhanced glycolytic metabolism, high intra-tumoral lactate levels and a worse anti-tumor immune response, associated with poor overall survival [3,7,41,42]. Therefore, metabolic characteristics of tumor cells and their influence on invading immune cells have created more interest in the past years.

Today it is clear that lactate and protons secreted by glycolytic tumor cells are not only a ‘waste product’ but rather have regulatory effects on invading immune cells. The negative impact of lactic acid on CD8 T cells and NK cells has been demonstrated by us and others in both human and murine CD8 T cells [6,16,18] but data are limited regarding the inhibitory function of lactic acid on CD4 T cells. Sodium lactate or lactic acid inhibit the proliferation of murine CD4 T cells [37,43] and Haas and colleagues have demonstrated that lactic acid treatment impairs the migration of human CD4 T cells in rheumatoid arthritis [44]. The same authors also showed that treatment of CD4 T cells with sodium lactate led to an altered cytokine secretion shifting the cells towards a Th17 phenotype [40].

To characterize the effects of lactic acid on CD4 T cells in more detail, we investigated the resistance of human bulk CD4 T cells and CD4 T cells sorted into naïve, central memory, and effector memory cells, respectively, against lactic acid treatment based on their viability, function and metabolism. We observed slight differences in cytokine production and viability, but based on all parameters tested no T cell subset displayed an overall higher lactic acid resistance. Lactic acid clearly diminished respiration and prevented the switch to glycolysis after T cell activation in all tested subsets. This metabolic blockade is most likely based on the uptake of lactate and protons via MCTs. This has two main consequences: first, the intracellular accumulation of lactate, and second, the acidification of the T cell cytoplasm, both of which interfere with different aspects of T cell function and metabolism as the activity of several enzymes is pH-dependent [45,46,47].

Different studies demonstrated that T cells isolated from human tumors displayed a decreased mitochondrial fitness [27,29], as intracellular acidification disturbed the mitochondrial membrane potential. It was also shown that the decrease in response of CLL patients to CAR T cell therapy was related to the degree of mitochondrial impairment of the infused CAR T cells [30]. This demonstrates that mitochondrial function contributes to T cell functionality in the tumor environment. Moreover, it suggests that cellular metabolism represents a potential target to increase lactic acid resistance of T cells. 

Therefore, we tried to overcome the impairment in T cell function by lactate and protons via metabolic reprogramming. Our hypothesis was that overexpression of LDHB might lead to metabolic conversion of intracellular lactate to pyruvate and thereby partially counteract the influx of lactate/protons in T cells as LDHB has a higher affinity for lactate compared to LDHA [31].

Analyzing lactate secretion and glucose tracing analysis revealed no significant changes in glucose metabolism of T cells with or without LDHB overexpression. Notably, lactic acid treatment resulted in a significantly reduced glucose uptake. Glucose flux in terms of degradation into pyruvate was unchanged. However, pyruvate conversion into lactate and TCA metabolites was diminished, indicating a block of pyruvate metabolism. Importantly, both enzymes involved, LDH and pyruvate dehydrogenase (PDH) have a pH optimum around 7.5, explaining their reduced activity upon lactic acid exposure, which causes a drop in intracellular pH below 7.0 [48,49]. 

The most pronounced effect of LDHB overexpression was an elevated oxygen consumption in the presence of lactic acid, whereas lactic acid treatment suppressed respiration in T cells with normal LDHB expression. LDHB-overexpressing cells displayed a higher respiratory activity under both control conditions and lactic acid treatment. The increased respiration was not accompanied by a higher mitochondrial content in LDHB overexpressing cells, suggesting an increased mitochondrial activity induced by LDHB overexpression and lactic acid treatment. As lactate oxidation by LDHB results in NADH generation, LDHB-overexpressing cells may have higher levels of NADH which could promote oxidative phosphorylation. The effect of LDHB overexpression on cellular respiration was even more pronounced upon lactic acid treatment. This could be a consequence from increased lactate conversion, glutaminolysis or fatty acid oxidation, all of which fuel TCA cycle activity [50]. More studies are necessary to elucidate the origin of increased cellular respiration of LDHB cells.

We also detected a higher percentage of cytokine-expressing cells among T cells with enhanced LDHB expression compared to those with normal LDHB levels. However, increased intracellular cytokine expression was only partially reflected by an increased cytokine secretion. A possible explanation could be the intracellular cytosolic acidification which may hamper trafficking of vesicles from the Golgi system to the cell surface and their exocytosis [51,52].

A shift of the metabolic preferences towards lactate consumption has been reported as a feature of murine Treg, and is also discussed as a possible mechanism allowing Treg to adapt to the tumor environment [38,40]. Moreover, it has been shown in murine CD4 T cells that the activity of the respiratory chain determined the cytokine responses [39]. This raised the possibility that overexpression of LDHB and manipulating cell metabolism could influence the differentiation of CD4 T cells and induce a regulatory phenotype. However, the frequency of Treg among the CD4 T cells was unchanged by LDHB overexpression, neither in human nor in murine T cells. In addition, the cytokine profile of the human LDHB overexpressing cells was not altered, demonstrating that the differentiation of CD4 T cells was not affected by LDHB overexpression.

Surprisingly, overexpression of Ldhb in murine T cells failed to upregulate respiration or cytokine expression upon lactic acid treatment excluding syngeneic murine tumor models to test the in vivo activity of LDHB-overexpressing T cells. Therefore, we decided to use spheroid co-culture experiments to further test the effects of LDHB overexpression in a physiological model. Spheroid co-culture experiments using colorectal carcinoma HCT116 spheroids revealed that LDHB-overexpressing cells preferentially migrated into spheroids and showed higher expression of cytotoxic effector molecules within the spheroids. This suggests, that LDHB overexpression in T cells could be beneficial for their anti-tumor activity and probably improve outcome of ACT treatment approaches. Notably, effects were most pronounced at 10 mM lactic acid treatment, suggesting that highly glycolytic tumors may respond worse to LDHB overexpressing T cells. Therefore, a combination of metabolic modification of T cells prior to ACT in combination with administration of low-dose glycolytic inhibitors to slightly reduce lactate levels could be a valuable strategy. 

The genetic modification of T cells before ACT with the overall goal to enhance their anti-tumor efficacy is already being applied in the clinics in terms of CAR T cell therapy [53]. Furthermore, metabolic manipulation of immune cells by genetic modification can enhance the anti-tumor efficacy of transferred T cells in murine tumor models and many of those attempts targeted the glucose and mitochondrial metabolism of the investigated cells. Scharping and colleagues demonstrated that overexpression of Pgc1a in murine T cells leads to enhanced mitochondrial biogenesis associated with an increased survival of tumor-bearing mice after ACT [27,28]. Aside, it was demonstrated that the deletion of Hif1a in T cells before transfer leads to an enhanced FAO activity and increases the anti-tumor activity of T cells [27]. Notably, HIF1a is a key transcription factor stabilized by hypoxia and upregulates the expression of key glycolytic enzymes, including LDHA. Therefore, it can be presumed that the deletion of HIF1a could have had a similar effect as we aimed by directly manipulating the balance between LDHA and LDHB. 

Overall, our data show that LDHB overexpression seems to represent a promising approach to increase the metabolic fitness of T cells for ACT but might have to be accompanied by additional modifications.

## 4. Materials and Methods

### 4.1. Human T Cell Isolation and Culture

Human T cells were isolated from MNCs of healthy donors after leukapheresis or directly from leukocyte reduction system cones followed by density gradient centrifugation over Ficoll/Hypaque. CD4 T cells were isolated by magnetic bead separation using Miltenyi CD4 Dynabeads (Miltenyi Biotec, Bergisch Gladbach, Germany). Purity was >98% as determined by flow cytometry in selected donors. All participants provided written informed consent and the study was approved by the local ethics committee (vote numbers 13-101-0240 and 13-101-0238).

T cells were cultivated in RPMI1640 (Thermo Fisher Scientific, Waltham, MA, USA) supplemented with 10% AB-serum (heat-inactivated; Bavarian red cross, Germany), 2 mM L-glutamine (PAN-Biotech, Aidenbach, Germany), 1 mM sodium pyruvate, essential vitamins, non-essential amino-acids, β-mercaptoethanol (50 µM), penicillin (50 IU/mL) and streptomycin (50 µg/mL) (all Thermo Fisher Scientific) in a humidified atmosphere (5% CO_2_, 95% air) at 37 °C in a Heraeus incubator (Thermo Fisher). For glucose restricted cultures, RPMI1640 without glucose, sodium pyruvate and non-essential amino acids (Biological Industries, Sartorius, Göttingen, Germany) was used. 

For experiments, 0.1 × 10^6^ cells were cultured in 96-well U-bottom plates in 225 µL T cell medium supplemented with 25 IU/mL (T cell memory subsets) or 50 IU/mL recombinant human IL-2 (Peprotech, Hamburg, Germany) and stimulated with anti-CD3/CD28 coated Dynabeads (Thermo Fisher Scientific) in a bead-to-cell ratio of 1:1. After 72 h, T cells were split and medium and IL-2 replenished. 

For determination of respiratory activity, 0.8 × 10^6^ T cells were seeded into OD24 Oxodishes (PreSens, Regensburg, Germany) in 1 mL final volume. Measurement was carried out using the PreSens HDR sensor dish system.

Lactic acid used in cell culture experiments was purchased from Sigma Aldrich (St. Louis, MO, USA).

### 4.2. Murine T Cell Isolation and Culture

Murine CD4 T cells were isolated from splenocytes of C57BL/6 mice (provided by the central animal laboratory facility, University of Regensburg) using magnetic cell separation by CD4 (L3T4) magnetic beads (Miltenyi Biotec) according to manufacturer’s instructions. 

Murine T cells were cultured in RPMI1640 supplemented with 10% FCS (heat-inactivated; Sigma-Aldrich), 2 mM L-glutamine, 1 mM sodium pyruvate, essential vitamins, non-essential amino acids, β-mercaptoethanol (50 µM), penicillin (50 IU/mL), and streptomycin (50 µg/mL) in a humidified atmosphere (5% CO_2_, 95% air) at 37 °C in a Heraeus incubator (Thermo Fisher). 

For experiments, 0.1 × 10^6^ cells were cultured in 96 well U-bottom plates in 200 µL T cell medium supplemented with 100 IU/mL recombinant IL-2 and 50 ng/mL IL-15 (Peprotech) and stimulated with the Treg activation and expansion kit (Miltenyi Biotec) in a bead-to-cell ratio of 1:1. 

For determination of respiratory activity, 1.2 × 10^6^ T cells were seeded into OD24 Oxodishes in 1 mL final volume. Measurement was carried out using the PreSens HDR sensor dish system.

### 4.3. Plasmids and Construct Cloning

For retroviral transfer into human T cells, coding sequence of the HIS-tagged human LDHB was cloned into the commercially available vector pMX-IRES-Puro via EcoRI and XhoI (New England Biolabs, Ipswich, MA, USA). For murine transduction, sequence of Ldhb was cloned into a modified MSCV-Thy1.1 vector using the Gateway technology. Bacterial amplification in XL1-Blue or TOP10 and purification of plasmids were performed according to manufacturer’s instructions (Qiagen, Hilden, Germany). All final plasmids were thoroughly sequenced (GeneArt, Regensburg, Germany). 

### 4.4. Generation LDHB Overexpressing Human T Cells

For retroviral transfer into human T cells, packaging cell line Platinum-A was used. Cells were cultured in DMEM (4.5 g/L glucose; Thermo Fisher Scientific) supplemented with 10% FCS (Sigma-Aldrich) and 2 mM L-glutamine in a humidified atmosphere (5% CO_2_, 95% air) at 37 °C in a Heraeus incubator. For virus generation, cells were transfected using the transfection reagent TransIT-LT (Mirus Bio, Madison, WI, USA) with the LDHB-containing or corresponding empty vector and the helper vectors pHIT60 and pCOLT-GALV. Virus supernatant was harvested and used 48 h after transfection or frozen at −80 °C.

CD4 T cells were isolated and preactivated for 48 h as described above. For transduction, stimulation beads were removed, and up to 4 × 10^6^ cells seeded into retronectin (Takara Bio Inc., Kusatsu, Japan) coated 12-well plates. Virus supernatant was added, spin transduction performed at 568 g, 30 °C, 90 min and cells incubated in a humidified atmosphere (5% CO_2_, 95% air) at 37 °C. Procedure was repeated once with fresh virus supernatant. 72 h after end of transduction, cells were treated with 5 µg/mL puromycin (Sigma-Aldrich) for selection. Afterwards, cells were expanded in 24-well plates by stimulation with anti-CD3/CD28 coated Dynabeads (bead-to-cell ratio 1:3) and 50 IU/mL IL-2. Cells were used for experiments 3–4 days after end of selection. Transduction and selection efficacy were monitored by flow cytometric analysis of HIS expression. 

### 4.5. Generation Ldhb Overexpressing Murine T Cells

For retroviral transfer into murine T cells, packaging cell line Phoenix-ECO was used. Cells were cultured in RPMI1640 supplemented with 10% FCS and 2 mM glutamine in a humidified atmosphere (5% CO_2_, 95% air) at 37 °C in a Heraeus incubator. For virus generation, cells were transfected with the Ldhb-containing or corresponding empty vector and the help vector pCL-Eco. 

For transduction, 0.04 × 10^6^ cells were seeded in 100 µL final volume in 96-well U bottom plates and stimulated using the Treg Activation and Expansion Kits (bead-to-cell ratio 1:1), 100 IU/mL recombinant IL-2 and 50 ng/mL IL-15. After overnight culture, 100 µL virus supernatant and polybrene (final concentration 3 µg/mL) were added. After 6 h, 150 µL were replaced with 160 µL fresh culture medium. Cells were used for experiments 72 h after end of transduction. Transduction efficacy was checked by flow cytometric analysis of CD90.1.

### 4.6. Co-Culture with HCT116 Spheroids

HCT116 tumor cell line was cultured in RPMI1640 supplemented with 10% FCS (heat-inactivated) and 2 mM glutamine in a humidified atmosphere (5% CO_2_, 95% air) at 37 °C in a Heraeus incubator. Spheroids were generated with the hanging-drop technique at a density of 0.01 × 10^6^ cells/30 µL. After 4–7 days, spheroids were harvested, washed, culture medium changed and transferred to 96-well plates coated with 1% agarose or 120 µg/mL poly-hema (Sigma-Aldrich) in a final volume of 200 µL.

Transduced human T cells were prestimulated with anti-CD3/CD28 coated Dynabeads in a bead-to-cell ratio of 1:1 at a density of 0.4 × 10^6^ cells/mL in 24-well plates in the presence of 50 IU/mL recombinant human IL-2. After 24 h, stimulation beads removed and 0.1 × 10^6^ cells/100 µL/well added to assembled spheroids by media exchange. After 48 h of co-culture, spheroids were treated with monensin for 3 h. Then, residual immune cells were washed away, spheroids lysed by incubation in trypsin/EDTA and infiltrated immune cells analyzed by means of flow cytometry.

### 4.7. Flow Cytometry

For purification of T cell memory subsets, human CD4 T cells were stained for CCR7CD45RO, CD62L (all BD Biosciences, Franklin Lakes, NJ, USA) and CD45RA (Miltenyi Biotec). Live, single cells were sorted into naïve (CD45RA^+^, CCR7^+^, CD62L^+^), central memory (CD45RO^+^, CCR7^+^, CD62L^+^) and effector memory (CD45RO^+^, CCR7^−^, CD62L^−^) T cells by four-way sorting on a BD FACSAria IIu. Purity was confirmed by re-analysis and sorted cells were further processed for experiments immediately.

Single cell suspensions from spheroid co-cultures were prepared as described above. For live-dead discrimination, cells were stained with 80 µL Zombie-NIR (diluted 1:650 in PBS; BioLegend, San Diego, CA, USA) for 10 min at RT in the dark. Afterwards, cells were stained for anti-CD45 and anti-CD4 (both BD Biosciences). Intracellular staining of IFN-γ, Granzyme-B (both BD Biosciences) and Perforin (eBioscience, San Diego, CA, USA) was performed for 20 min after lysis using the BD Cytofix/Cytoperm kit (BD Biosciences) according to the manufacturer’s protocol.

For determination of intracellular cytokine production of transduced T cells, murine and human T cells were pre-treated with the respective conditions over night as described above and stimulated for 3–4 h with PMA and Ionomycin in the presence of monensin.

Human T cells were stained with anti-HIS (Miltenyi Biotec), anti-TNF (BioLegend) and anti-IFN-γ using the BD Cytofix/Cytoperm kit. Murine T cells were stained with Zombie-NIR for live-dead discrimination, anti-CD3, and anti-CD4. Intracellular staining for Ifn and Tnf was performed using the BD Cytofix/Cytoperm kit (all antibodies from BioLegend).

For estimation of regulatory T cell portion, human and murine T cells were stained for human and murine CD25 and Foxp3, respectively. Foxp3 staining was performed using the Intracellular Fixation and Permeabilization buffer set (Thermo Fisher Scientific) according to manufacturer’s instructions.

For estimation of mitochondrial content, human LDHB-overexpressing T cells were stained with Mitotracker Green FM (Thermo Fisher Scientific) three days after end of selection. Therefore, 0.5 × 10^6^ cells were suspended in 1 mL T cell medium without AB-serum, treated with 10 nM Mitotracker Green FM and 1.3 µm Cyclosporine A (Sandimmun^®^, Novartis, Basel, Switzerland) and incubated under cell culture conditions for 1 h.

Apoptosis was determined by Annexin-V/7-AAD staining (BD Biosciences).

All measurements were carried out using the BD FACS Calibur, BD LSR Fortessa or the BD FACS Celesta. Data were analyzed with the FlowJo software (v10.6.2; Tree Star, Ashland, OR, USA).

### 4.8. Preparation of RNA, Reverse Transcription, and Quantitative Real-Time PCR

Purification of RNA, Reverse Transcription and quantitative Real-Time PCR for gene expression analysis in transduced T cells was performed as described elsewhere [54]. Primer sequences (all from Eurofins MWG Operon, Ebersberg, Germany) were as follows (-5′-3′); (F-Forward; R-Reverse): 18s rRNA F-ACCGATTGGATGGTTTAGTGAG, R-CCTACGGAAACCTTGTTACGAC; LDHA F-GGTTGGTGCTGTTGGCATGG, R-TGCCCCAGCCGTGATAATGA; LDHB F-GATGGTGGTTGAAAGTGCCTATGAAGTC, R-AGCCACACTTAATCCAATAGCCCA.

### 4.9. Preparation of Whole Cell Lysates, Western Blotting and LDH Isoenzyme Determination

Whole cell lysates were prepared using RIPA buffer (Sigma-Aldrich) or Cell Lysis Buffer (1×; Cell Signaling Technology, Danvers, MA, USA) and quantified with the Qubit Protein Assay Kit (Thermo Fisher Scientific).

After blotting, membranes were stained with anti-LDHA/Ldha (Cell Signaling Technology), anti-LDHB/Ldhb (Santa Cruz Biotechnolgy, Dallas, TX, USA) or anti-Actin (Sigma Aldrich) and analyzed using the chemiluminescence system Fusion Pulse 6 (Vilber Lourmat, Eberhardzell, Germany).

For LDH isoenzyme determination, only lysates in Cell Lysis Buffer were used. Analysis was carried out using the SAS-MX CK/LD Vis Kit or the Quickgel LD QG Kit (Helena Biosciences, Gatesham, United Kingdom). Samples were thawed on ice, diluted in PBS to equal concentrations and loaded to the gels after manufacturer’s instructions. Gel run was carried out at 100 V (SAS-MX) for 15 min and 25 min or 400 V (Quickgel) for 5 min and 8 min for human and murine samples, respectively. Detection of Isoenzymes was performed according to manufacturer’s instructions at 45 °C. Afterwards, gels were destained by washing in 1 M acetic acid and washed in distilled water. As a positive control for murine analyses, whole kidney lysates were used. Pictures were recorded using the Fusion FX Imaging System (Vilber Lourmat).

Band intensities of the LDH isoenzyme staining was quantified using ImageJ according to a protocol published by Hossein Davarinejad [55]. Values from LDHB overexpressing cells were normalized on respective Mock values.

### 4.10. ELISA

IFN-γ, TNF, IL-10, IL-17, Ifn-γ and Tnf were measured by ELISA (all Kits purchased from R&D systems, Minneapolis, MN, USA). Cytokines were either measured in cell culture supernatants after anti-CD3/CD28 stimulation or after stimulation with PMA/Ionomycin as given in the respective figure legends.

### 4.11. Determination of Cell Number

Cell proliferation in culture was monitored using the CASY Cell Counter system.

### 4.12. Quantification of Lactate Secretion and Glucose Consumption

Lactate and glucose were measured in culture supernatants of T cells. Lactate concentration was measured at the Institute of Clinical Chemistry at the University Hospital Regensburg.

Glucose levels were measured using the Glucose (HK) assay kit (Sigma-Aldrich) according to manufacturer’s instructions. Glucose uptake was calculated by subtracting the remaining glucose concentration from the basal medium glucose concentration.

### 4.13. siRNA Mediated Knockdown of LDHA

The siRNAs targeting LDHA and the respective control pool were achieved from Dharmacon (Lafayette, DA, USA) and dissolved in PCR-grade H_2_O to a final concentration of 1 µg/µL and stored at −20 °C.

Electroporation with siRNAs was performed with unstimulated CD4 T cells directly after isolation. A total of 1 × 10^7^ cells per electroporation were washed first with RPMI1640 without phenol red and then with 10 mL OptiMEM (Thermo Fisher Scientific). Afterwards, cells were suspended in OptiMem to a final concentration of 5 × 10^7^ cells/mL. Electroporation with siRNA was performed with 1 µg siRNA/1 × 10^7^ cells. Cells were directly transferred to 4 mL pre-warmed cell culture medium, rested overnight and afterwards stimulated for experiments.

### 4.14. ^13^C-Glucose Tracing

To perform ^13^C_6_-glucose tracing in LDHB overexpressing T cells, cells were washed with glucose-free medium 72 h after end of selection and re-stimulated with anti-CD3/CD28 Dynabeads (ratio 1:5) in the presence of 10 mM ^13^C_6_-glucose (Cambridge Isotope Laboratories, Tewksbury, MA, USA) with or without lactic acid. Cells were cultured for 6 h in the presence of the indicated compounds, washed 2× in PBS, and immediately frozen in liquid nitrogen. Cell pellets were kept at −80 °C until further analysis. Experiments were performed with cells from 3 different donors. Metabolite extraction and GC-MS were performed as described previously [23].

## Supplementary Materials

The following supporting information can be downloaded at: https://www.mdpi.com/article/10.3390/ijms23115970/s1.
