# Supplementary material for: LDHB Overexpression Can Partially Overcome T Cell Inhibition by Lactic Acid
Source: Int J Mol Sci. 2022 May 26;23(11):5970. doi: 10.3390/ijms23115970 (PMC9180663; doi:10.3390/ijms23115970)
Supplement: Supplementary file 1 [file ijms-23-05970-s001.zip › ijms-1716721-supplementary.pdf]

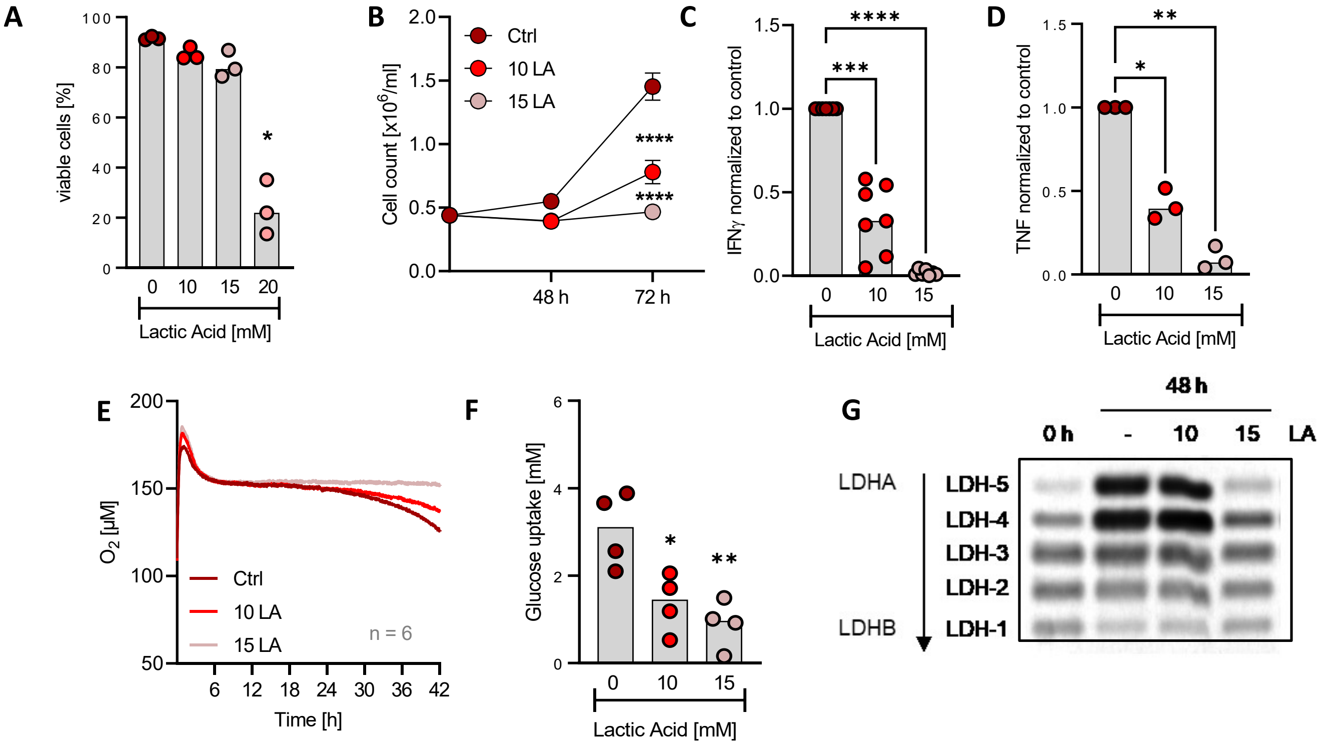

Figure S1. Lactic acid inhibits bulk CD4 T cell effector functions by metabolic blockade. (**A – G**) CD4 T cells were stimulated with anti-CD3/CD28 coated beads (bead-to-cell ratio 1:1) and treated with the indicated concentrations lactic acid in standard RPMI. (**A**) Viability was determined after 72 h by annexin V/7-AAD staining. Shown are median values and single data points. Statistical significance was calculated using one-way ANOVA and Bonferroni multiple comparison test (* $p < 0.05$). (**B**) Cells were counted at indicated timepoints using the CASY Cell Counter (mean + SEM, n = 4). Statistical significance was calculated with two-way ANOVA and Dunnet's multiple comparison test (* $p < 0.05$; **** $p < 0.001$). (**C, D**) Cytokine concentrations in supernantants were determined after 48 h using ELISA and normalized to the respective controls. Shown are median values and single data points. Statistical significance was calculated using one-way ANOVA and Bonferroni multiple comparison test (* $p < 0.05$, ** $p < 0.01$, *** $p < 0.001$). (**E**) Oxygen consumption of the cells was measured using the PreSens technology (mean values, n = 6). (**F**) Glucose uptake was calculacted by substracting remaining glucose in culture supernants after 48 h from basal glucose concentration in culture medium. Shown are median values and single data points. Statistical significance was calculated using one-way ANOVA and Bonferroni multiple comparison test (* $p < 0.05$, ** $p < 0.01$). (**G**) LDH isoenzyme distribution in unstimulated (0 h) and 48 h stimulated and lactic acid treated cells was assessed using LHD zymography analysis. Depicted is one representative example (n = 3).

Figure S2

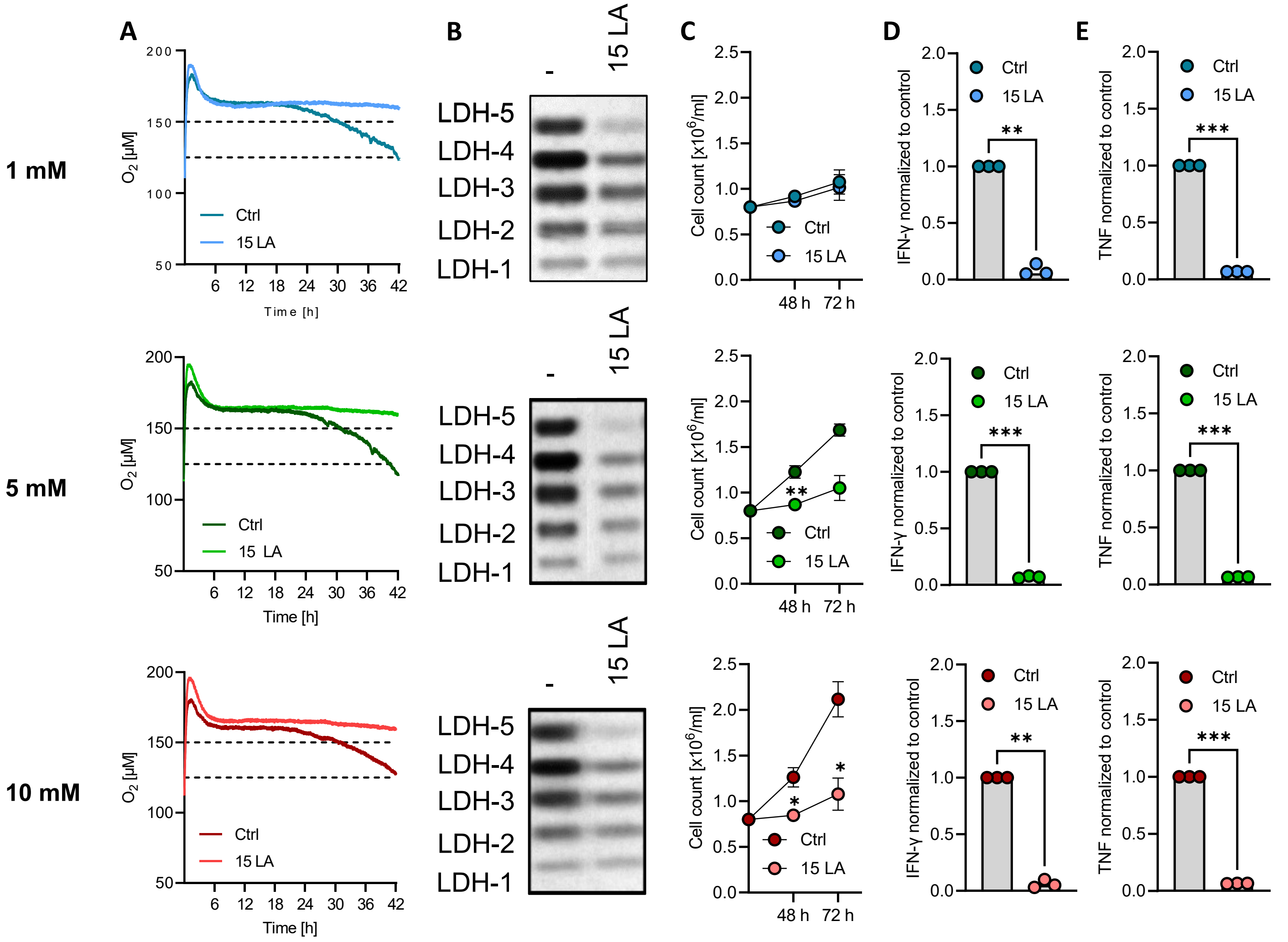

Figure S2. Lactic acid inhibits bulk CD4 T cell effector functions by metabolic blockade independent of glucose supplementation. (**A – E**) CD4 T cells were stimulated with anti-CD3/CD28 coated beads (bead-to-cell ratio 1:1) and treated with the indicated concentrations lactic acid in RPMI supplemented with given glucose concentrations. (**A**) Oxygen consumption of the cells was measured using the PreSens technology (mean values, n = 6). (**B**) LDH isoenzyme distribution in unstimulated 48 h stimulated and lactic acid treated cells was assessed using LHD zymography analysis. Depicted is one representative example (n = 3). (**C**) Cells were counted at indicated timepoints using the CASY Cell Counter (mean + SEM, n = 4). Statistical significance was calculated with two-way ANOVA and Dunnet's multiple comparison test (* $p < 0.05$; ** $p < 0.01$). (**D, E**) Cytokine concentrations in supernantants were determined after 48 h using ELISA and normalized to the respective controls. Shown are median values and single data points. Statistical significance was calculated using one-way ANOVA and Bonferroni multiple comparison test (** $p < 0.01$, *** $p < 0.001$).

Figure S3

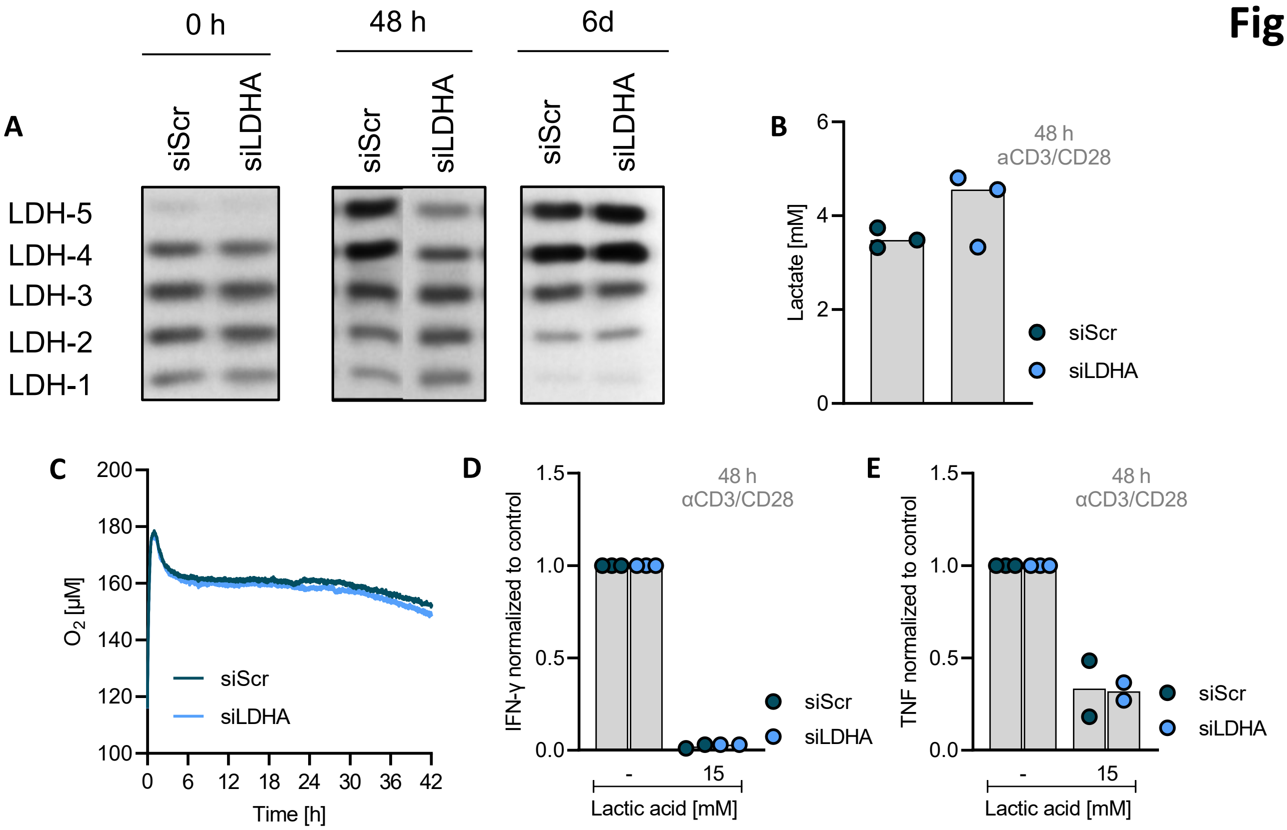

Figure S3. LDHA knockdown in T cells does not increase lactic acid resistance. CD4 T cells were electroporated with siRNA targeting LDHA (siLDHA) or a scrambled control (siScr). After resting over night, cells were stimulated with anti-CD3/CD28 coated beads (bead-to-cell ratio 1:1) and treated with 15 mM lactic acid. (**A**) LDH isoenzyme distribution in unstimulated (0 h) and 48 h stimulated and lactic acid treated cells was assessed using LHD zymography analysis. Depicted is one representative example (n = 3). (**B)** Lactate concentration in culture supernants was assessed enzymatically after 48 h stimulation. (**C**) Oxygen consumption of the cells was measured using the PreSens technology (mean values, n = 3). (**D, E**) Cytokine concentrations in supernantants were determined after 48 h using ELISA and normalized to the respective controls. Shown are median values and single data points. (**B, D, E**) Statistical significance was calculated using one-way ANOVA and Bonferroni multiple comparison test.

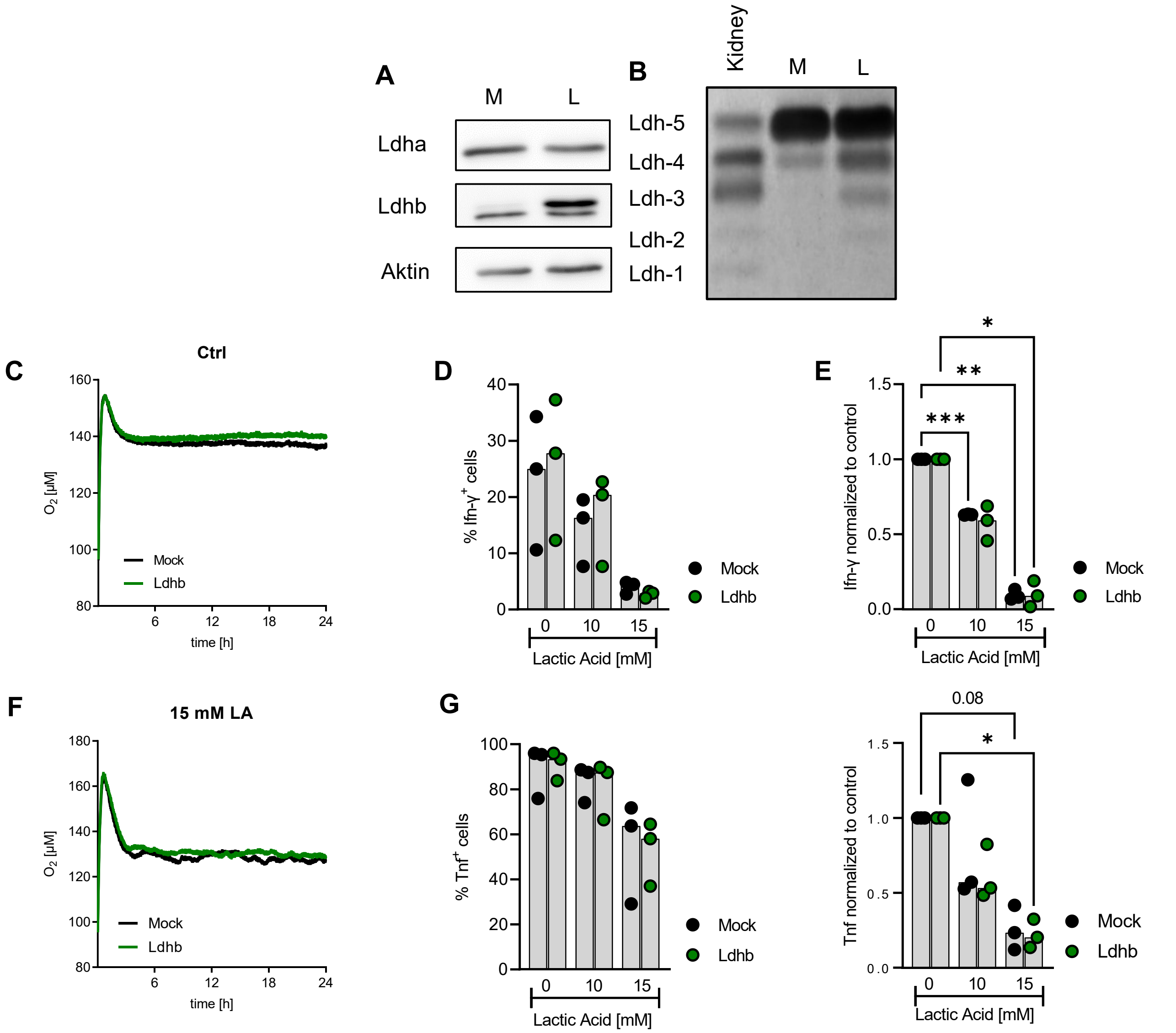

Figure S4. Overexpression of Ldhb in murine T ells fails to alter cellular respiration or increase lactic acid resistance. Ldhb was overexpressed in murine CD4 T cells. (**A**) Expression of Ldha and Ldhb was assessed by western blot analysis. Actin served as a loading control. Depicted is one representative example (M: Mock, L: Ldhb; n = 3). (**B**) LDH isoenzyme distribution was assessed using LHD zymography analysis. Depicted is one representative example (M: Mock, L: Ldhb; n = 3). (**C**) Cellular oxygen consumption under control conditions and upon 15 mM lactic acid treatment was measured using the PreSens technology (mean values, n = 3). (**D**) Intracellular production of Ifn-γ and Tnf was analyzed after stimulation with PMA/Ionomycin and monensin treatment. (**F**) Cytokine concentrations in supernantants were determined after PMA/Ionomycin stimulation using ELISA and normalized to the respective controls. (**D – G**) Shown are median values and single data points. Statistical significance was calculated using one-way ANOVA and Bonferroni multiple comparison test (* $p < 0.05$, ** $p < 0.01$, *** $p < 0.001$).
